# Examining within- and across-day relationships between transient and chronic stress and parent food-related parenting practices in a racially/ethnically diverse and immigrant population: Stress types and food-related parenting practices

**DOI:** 10.1186/s12966-017-0629-1

**Published:** 2018-01-16

**Authors:** Jerica M. Berge, Allan Tate, Amanda Trofholz, Angela Fertig, Scott Crow, Dianne Neumark-Sztainer, Michael Miner

**Affiliations:** 1 Department of Family Medicine and Community Health, University of Minnesota Medical School, 717 Delaware Street SE, Room 425, Minneapolis, MN 55414 USA; 2 University of Minnesota, Humphreys Institute, Minneapolis, USA; 3 Department of Psychiatry, University of Minnesota, Minneapolis, MN USA; 4 The Emily Program, St. Paul, MN USA; 5 University of Minnesota, Division of Epidemiology and Community Health, Minneapolis, USA

**Keywords:** Transient stress, Chronic stress, Minority stress model, Parent feeding practices

## Abstract

**Background:**

Although prior research suggests that stress may play a role in parent’s use of food-related parenting practices, it is unclear whether certain types of stress (e.g., transient, chronic) result in different food-related parenting practices. Identifying whether and how transient (i.e., momentary; parent/child conflict) and chronic (i.e., long-term; unemployment >6 months) sources of stress are related to parent food-related parenting practices is important with regard to childhood obesity. This is particularly important within racially/ethnically diverse parents who may be more likely to experience both types of stress and who have higher levels of obesity and related health problems. The current study examined the association between transient and chronic stressors and food-related parenting practices in a racially/ethnically diverse and immigrant sample.

**Methods:**

The current study is a cross-sectional, mixed-methods study using ecological momentary assessment (EMA). Parents (mean age = 35; 95% mothers) of children ages 5–7 years old (*n* = 61) from six racial/ethnic groups (African American, American Indian, Hispanic, Hmong, Somali, White) participated in this ten-day in-home observation with families.

**Results:**

Transient stressors, specifically interpersonal conflicts, had significant within-day effects on engaging in more unhealthful food-related parenting practices the same evening with across-day effects weakening by day three. In contrast, financial transient stressors had stronger across-day effects. Chronic stressors, including stressful life events were not consistently associated with more unhealthful food-related parenting practices.

**Conclusions:**

Transient sources of stress were significantly associated with food-related parenting practices in racially/ethnically diverse and immigrant households. Chronic stressors were not consistently associated with food-related parenting practices. Future research and interventions may want to assess for transient sources of stress in parents and target these momentary factors in order to promote healthful food-related parenting practices.

## Background

Disparities in childhood obesity continue to be evident, with low-income and minority children having the highest prevalence of obesity [1–5]. Prior studies have shown that parents who use certain food-related parenting practices such as, restriction and pressure-to-eat feeding practices [6–11] and serving less healthful foods at meals (e.g., fast food) [12–16] have children who are at increased risk for overweight/obesity. However, factors that influence the use of these food-related parenting practices are not well understood. Identifying such factors is crucial for developing interventions to reduce the use of unhealthy food-related parenting practices to thereby decrease childhood obesity.

Research suggests that stress may play a role in parents’ use of unhealthy food-related parenting practices (e.g., controlling feeding practices, serving fast-food for meals) [17, 18]. However, it is unclear whether certain types of stress (e.g., chronic, transient) result in different food-related parenting practices [19, 20]. Chronic stressors are longer-lasting sources of stress (e.g., unemployment >6 months) whereas, transient stressors are temporary and more quickly resolved sources of stress (e.g., momentary conflict with child) [19]. For example, a family experiencing unemployment or chronic illness of a family member may experience high levels of chronic stress that remains constant over days, weeks, or months. On the other hand, stress experienced after a difficult encounter with a child around picky eating (transient stress) in the morning may affect evening feeding practices within day (or between days), but may not maintain across time. Distinguishing between transient and chronic stress in minority and immigrant households would be important because they may be more likely to experience both types of stress, which could put them at higher risk for engaging in restriction and pressure-to-eat feeding practices or reverting to feeding their family fast food. For example, minority and immigrant households may experience transient stressors such as financial stress (e.g., housing issues, running out of public assistance before the end of the month), while at the same time experiencing ongoing chronic stressors such as unemployment. Research utilizing longitudinal and time-lagged data is needed to examine the association between parental sources of stress and food-related parenting practices within-day (i.e., same day) and across-day (i.e., across the week) [21–24].

Prior research examining stress and food-related parenting practices has not utilized methods that are sensitive to within-day and across-day variations, such as ecological momentary assessment (EMA). Examining momentary stress within- and across-day is critical in obtaining a refined view of the home environment to identify factors that may explain why there are disparities in childhood obesity among racial/ethnic and immigrant groups. Designs that incorporate EMA analyses also address limitations with cross-sectional designs, such as reverse causality. EMA data can distinguish the temporal ordering of variables, such as transient and chronic stress and subsequent parent feeding practices over time. Additionally, prior research has not differentiated between transient and chronic stress, which has held the field back in understanding the relationship between stress and food-related parenting practices in minority and immigrant populations.

The current study is guided by the Minority Stress Model [20]. According to the Minority Stress Model, minority stress is “the excess stress (i.e., chronic) to which individuals from stigmatized social categories are exposed, often as a result of their minority position” [20, 25–27]. Research supports this tenet and shows that chronic stress experienced by racial/ethnic and immigrant populations is linked to poorer health outcomes (e.g., obesity, substance-abuse, depression) [26, 28]. Additionally, the Minority Stress Model posits that both transient and chronic stress are linked to the expression of maladaptive behavior(s) (e.g., restriction, pressure-to-eat feeding practices). Thus, it is important to include both measures of chronic (e.g., stressful life events) and transient (e.g., a lot of things to get done) stress in examining their influences on food-related parenting practices in racially/ethnically diverse and immigrant populations.

In order to advance the state of the field with regard to transient and chronic stress and parent food-related parenting practices within racially/ethnically diverse and immigrant populations, the main aims of the current study are to: [1] identify the effects of transient and chronic stress on daily parental stress levels in a racially/ethnically diverse and immigrant population; [2] examine how transient stressors and chronic stress levels are related to food-related parenting practices (i.e., parent feeding practices, types of food served at meals, food categories served at meals); and [3] evaluate whether transient sources of stress have persistent, weak, or strong effects on food-related parenting practices over time. Our main hypothesis is that transient stressors will be more strongly associated with parent food-related parenting practices compared to chronic stressors and food-related parenting practices.

## Methods

Data for the current study are from *Family Matters* [29], a 5-year incremental (Phase *I* = 2014–2016.; Phase II = 2017–2019) mixed-methods (e.g., video-recorded tasks, EMA, interviews, surveys) study designed to identify novel risk and protective factors for childhood obesity in the home environments of racially/ethnically diverse and primarily low-income children. Phase I includes an in-depth mixed-methods cross-sectional examination of the family home environment of diverse families (*n* = 150). Phase II involves a longitudinal epidemiological cohort study with diverse families (*n* = 1200). Additional details regarding the study design for both Phases of the study can be found elsewhere [29].

Data for the current study are from Phase I of the *Family Matters* study. In Phase I, a mixed-methods analysis of the home environments of children ages 5–7 years old from six racial/ethnic groups including, African American, American Indian, Hispanic/Latino, Hmong, Somali, and White (*n* = 25 from each racial/ethnic group) was conducted to identify individual, dyadic, and familial risk and protective factors for childhood obesity. The University of Minnesota’s Institutional Review Board Human Subjects Committee approved all protocols used in both phases of the *Family Matters* study.

### Recruitment and data collection

Children (*n* = 150) and their families were recruited from the Minneapolis/St. Paul, metropolitan area in Minnesota between 2015 and 2016 via a letter sent to them by their family physician. The sample was intentionally stratified by race/ethnicity and weight status (overweight/obese = BMI ≥85%ile; non-overweight = BMI >5%ile and <85%ile) of the child to identify potential weight- and/or race/ethnic-specific home environment factors related to obesity risk. A 10-day in-home observation was conducted with each family, including two in-home visits and an 8-day direct observational period in between home visits. The observational components included parent and child accelerometry, child dietary recalls, an interactive observational family task, and ecological momentary assessment (EMA) [29].

#### Sample demographics

The current analytic sample included 61 parent/child dyads (from the original 150 sample) with consecutive EMA data provided by the primary caregiver during the 8-day in-home observation period. The sample included families who were equally distributed across the six racial/ethnic groups recruited in the study (African American, American Indian, Hispanic, Hmong, Somali, White). Additionally, families were from low-income households, with 70% of families earning less than $35,000 per year. The majority of participants were mothers (95%) who were approximately 35 years old (mean = 34.9; sd = 7.1) with children ages 6 years old (mean = 6.5; sd = 0.08). Over half of the mothers worked full or part-time and almost 60% had a high school diploma or less. About half of the mothers were married and over 60% of households had two parents. The sub-sample demographics were almost identical to the full sample demographics [30].

### Measures

#### Ecological momentary assessment (EMA)

Multiple daily measures of EMA over 8 days were collected on parents. Standardized EMA data collection protocols from prior studies [31] were used in the study including: [1] signal contingent, [2] event contingent, and [3] end-of-day EMA messaging [31]. iPad minis were provided to parents to enter responses to the EMA surveys during the eight-day observation period.

Signal contingent recordings were researcher-initiated and were used in a stratified random manner so that each parent was prompted via a text message to fill out a survey four times a day, within a three-hour time block (e.g., 7–10 am, 11–2 pm, 3 pm–6 pm, 7–10 pm). The timing of EMA prompts was adjusted for parent shift work and wake times to accommodate parent’s differing life situations. The signal contingent recordings allowed for examining different contexts that occurred day-to-day, moment-by-moment, in families’ lives. Event contingent recordings were self-initiated by parents whenever an eating occasion (i.e., child and at least one other person were eating) occurred with the child. Parents were asked to fill out information about food preparation and meal planning, the logistics of the meal (e.g., who was there, type of food served, what the child ate), and behaviors occurring at the meal (e.g., parent feeding practices, child eating behaviors, meal atmosphere). The end-of-day recording was completed prior to sleep to capture any events not reported since the last recording, and to get end-of-day measures.

EMA compliance was high, with100% of participants completing all 8 days of EMA data collection and meeting the daily minimum requirement (i.e., 2 signal contingent, 1 event contingent, 1 end-of-day contingent; total = 4 EMA responses/day). On average, participants completed 7.4 surveys per day. Other details regarding the EMA component of the study have been published elsewhere [29, 30].

#### Parent transient stress/sources of stress

Parental stress was measured via multiple signal contingent EMA surveys throughout the day using items adapted from the Daily Health Diary (i.e., How stressed are you feeling right now?) [32]. At the end of the day, parents were asked about their overall stress from the day (i.e., Overall, how stressful was your day?). Response options ranged from “not at all” to “extremely” (range 0–4). In addition, parents were asked about their main source of stress over the day (i.e., Overall, what caused you the most stress today?). There were five response options that were categorized into three related sources of stress for analysis. Category #1 “A lot of work at home, school, or job” combined response options one and two: a lot of work to get done at job or school and many things to get done at home. Category #2 “Conflicts with spouse, partner, or children” combined response options three and four: conflicts or arguments with my spouse or romantic partner and conflicts with children or having to discipline. Category #3 “Financial problems” included response option five: worry about money/financial problems [32]. These three categories were used to characterize transient stressors as a dichotomous random variable.

#### Parent chronic stress/sources of stress

Overall chronic stress was measured using a self-report survey item [32]. Parents were asked on a scale of 1 to 10 how stressed they were over the last 30 days. A dichotomous variable was created and cut at a parent report of six or higher to represent elevated chronic stress. In addition to the overall chronic stress summary measure, parents’ report of stressful life events over the last 6 months from the brief life events questionnaire [33] (e.g., divorce, death of a family member, unemployment, problems with the police, serious illness), were measured as sources of chronic stress [34].

The twelve stressful life events items [33] were further defined as: 1) strong, active event (“Yes, and I still think about it a lot”), 2) weak, active event (“Yes, and I still think about it a little”), 3) resolved, inactive event (“Yes, but I do not think about it”), and 4) no stressful life event occurred (i.e., the parent had not experienced the event). Two stressful life events indicator variables (i.e., two “present/absent” dichotomous categorical variables) were constructed from the four response options, indicating the parent had experienced at least one strong/weak event that they still thought about and at least one stressful life event that was now resolved (i.e., the parent did not think about anymore). Parents that experienced neither an active or a resolved life event were represented as the reference value for the two indicator variables.

Acculturation status was also considered an important chronic stress for immigrant participants. Seven items from the AHIMSA-8 scale [35] were used to measure participant assimilation, separation, and integration. Parents reported on these three constructs by identifying which country they identified with: 1) United States for assimilation; 2) “The country my family is from” for separation; and 3) both for integration. Items included “I am most comfortable being with people from…” and “the food I eat at home is from…” to name two. Scales were scored by counting the number of response values corresponding to assimilation, separation, and integration.

#### Parent feeding practices

Parent restriction and pressure-to-eat parent feeding practices were measured during event contingent (i.e., meal occasions) EMA surveys using two items modeled after the Child Feeding Questionnaire [36]. Parent restriction (i.e., Did you have to make sure [child’s name] didn’t eat too much food at this meal?) and pressure-to-eat (i.e., Did you have to encourage [child’s name] to eat more food at this meal?) feeding practices at meal occasions were measured as a dichotomous variable (0 – “No”, 1 – “Yes”). To characterize daily feeding practices, the fraction of daily meals during which parents reported using each practice was calculated to represent the composition of feeding practices used by the parents over the course of the day. For example, if a participant reported pressuring feeding practices at two of the three meal occasions occurring during the day, the binomial outcome variable at the day level would be equal to 0.667 (i.e., 2 meal occasions in which the feeding practice was observed divided by the 3 total meals occurring that day). If the parent reported no pressuring at any of the three meals, then the variable would be equal to zero for that day.

#### Food types served at meals

The type of foods served at meal occasions were assessed during event contingent EMA surveys. Parents were asked what types of foods were served at the meal (i.e., Which best describes the type of food served?), adapted from prior survey research questions [15, 37]. The response options for types of food served included: fast food, homemade, or pre-prepared foods. Parents could report multiple types of foods served at any one meal occasion. The fraction of evening meal occasions (at the participant, observation day level) in which the food type was present was operationalized as a binomial outcome variable in the same way as the parent feeding practices variable (i.e., range 0–1). Like the example provided above, if the daily proportion of fast food at daily meal occasions was equal to 0.667, then two-thirds of the daily meal occasions had a fast food item present.

#### Foods served at meals

Parents were asked which foods were served at the meal, based on a pre-existing measure of meal healthfulness [16]. Parents could select all the food categories that applied. Options included: fruits, vegetables, whole grains (e.g., whole wheat breads or cereals, brown rice, oatmeal, corn tortillas), refried grains (e.g., white bread or cereals, flour tortillas, white rice), dairy, meat proteins, beans/eggs/seeds, sugary drinks (SSBs), cakes/cookies, or candy. Dummy variables were generated to characterize the composition of foods served and collapsed into five categories: 1) desserts, candy, and SSBs, 2) fruits and/or vegetables, 3) meat or plant proteins, 4) any grains, and 5) dairy. The fraction of daily meals in which these foods were present was calculated using the method described above for the fast food, pre-prepared, and homemade food types variables.

### Statistical analysis

A series of descriptive and time series analyses were performed to identify a study subsample (*N* = 61 of the *N* = 150 total participants) in which participants had consecutive daily EMA surveys (e.g., participants had no gaps in their 8-day EMA observation). For the 61 participants, a total of *N* = 383 observation days were available for analysis. Stress autocorrelations across observation days were evaluated to determine the maximum number of lagged variables to be included in the models. A model with two lags (i.e., labeled “L.1” and “L.2” for each daily source of stress) was retained based on statistically significant relationships with several sources of stress which were persistent through the second lag. For example, the interpretation of the L.2 coefficient is the association between the source of stress observed on a given day on the dependent variable observed 2 days later. Because eight consecutive days are available and a two-day lag model was fit, the first 6 days of sources of stress and all eight dependent variable observations are evaluated (e.g., L2 is not estimable on day seven because a ninth observation day would be needed for the dependent variable). The within-day association (i.e., cross-sectional relationship) was labeled “L.0.” In addition to the chronic and transient stress measures described above, each model adjusted for parent age, sex, race, country of origin, relationship status, immigrant acculturation status (assimilation, separation, and integration) [35], and day of the week. General linear mixed models were used to evaluate outcomes including stress, parent food preparation, and feeding practices as continuous random variables. All independent predictors for which coefficients are presented were evaluated as categorical variables. A random intercept at the participant level, a random slope for the observation day (i.e., time), and an unstructured covariance structure was specified to account for the longitudinal design. All statistical analysis was performed in Stata 15 SE (College Station, TX).

## Results

### Descriptive findings for transient stress levels across the week and chronic stress by racial/ethnic and immigrant groups

**Fig. 1:**
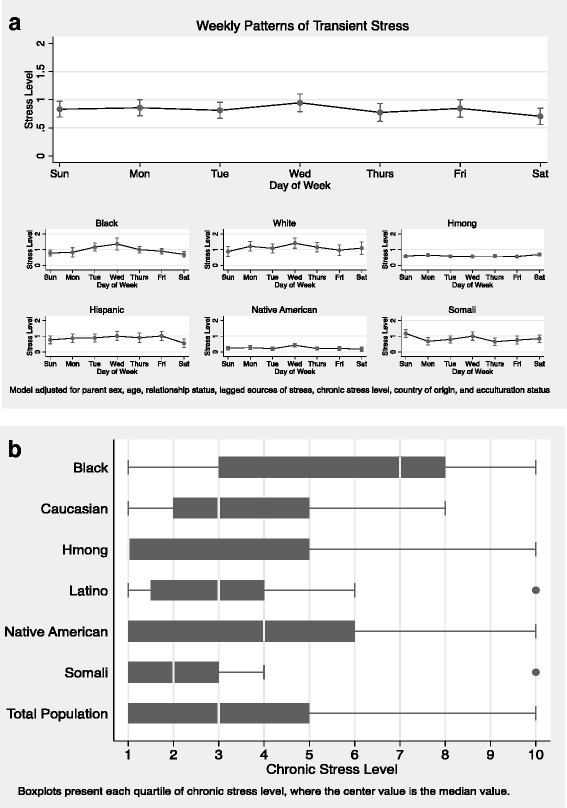
**a**-**b** Patterns of Transient and Chronic Stress by Race/Ethnicity. **a** Predicted Daily Transient Stress Patterns. **b** Chronic Stress Level Boxplots Stratified by Race Group

Transient stress level patterns in the sample were low overall and increased from the beginning of the week until they peaked in the middle of the week (i.e., Wednesday) and then decreased again throughout the rest of week (Fig. 1a). Within racial/ethnic and immigrant groups, White, African American, Hispanic and Somali parents had similar patterns to the overall sample’s transient stress pattern. Whereas, Hmong, and Native American parents had low and flat transient stress levels throughout the week.

The average chronic stress levels in the sample were low to moderate (3.6 ± 2.8, Fig. 1b). Within racial/ethnic and immigrant groups, African American and Native American parents had the highest chronic stress levels (5.6 ± 3.2 and 4.3 ± 3.4 respectively). White (3.6 ± 2.1), Hispanic (3.3 ± 2.4), and Hmong (3.1 ± 3.4) parents had chronic stress levels about the sample average, and Somali parents (2.5 ± 2.5) reported the lowest levels of chronic stress.

### Relationship between transient and chronic stress and end-of-day stress levels

Within-day relationships between the sources of transient stress (i.e., a lot of work to get done at home, school, or job; interpersonal, financial) and end-of-day stress level was highly significant (*p* < 0.01; Table 1). When examining specific sources of stress, interpersonal conflicts had the strongest within-day effect on end-of-day stress levels (1.45; 95% CI: (1.25, 1.64)) followed by many things to get done at work, home, or school (1.28; 95% CI: (1.11, 1.45)). The strength of the across-day association weakened by day two for both of these sources of stress and by day three the relationship was not found to be statistically significant. Transient stress due to financial problems had the weakest effect on end-of-day stress level (0.71; 95% CI: (0.32, 1.11), *p* < 0.001), but the relationship was the most persistent across the 3 days.

**Table 1:** Effects of chronic and lagged transient stressors on end-of-day stress over three days (*N*=61 participants; 383 observation days)^a^

Independent predictor variable	Mean response	95% CI	*P* value^b^
**Chronic stressors**			
*High chronic stress indicator (ref: Low chronic stress)*^*c*^	**0.61**	**(0.30, 0.93)**	**0.000**
*Stressful life events (prior 6 months)*			
Active (*ref: no active events*)	0.18	(-0.06, 0.42)	0.138
Resolved (*ref: no resolved events*)	-0.07	(-0.28, 0.14)	0.504
**Transient stressors**			
*Lagged sources of stress*			
*"A lot of work at home, school, or job"*			
L0. (same day)	**1.28**	**(1.11, 1.45)**	**0.000**
L1. (second day)	**0.25**	**(0.09, 0.41)**	**0.003**
L2. (third day)	0.13	(-0.03, 0.29)	0.116
*"Conflicts with spouse, partner, or children"*			
L0. (same day)^c^	**1.45**	**(1.25, 1.64)**	**0.000**
L1. (second day)	0.17	(-0.03, 0.36)	0.091
L2. (third day)	-0.02	(-0.21, 0.17)	0.800
*"Financial problems"*			
L0. (same day)	**0.71**	**(0.32, 1.11)**	**0.000**
L1. (second day)	**0.77**	**(0.33, 1.21)**	**0.001**
L2. (third day)	**0.57**	**(0.14, 1.00)**	**0.009**

^a^Model adjusted for: Parent sex, age, race, country of origin, relationship status, acculturation status (assimilation, separation, and integration), and day of the week

^b^Boldface values indicate statistical significance at *p* < 0.05

^c^ *Interpretation Example: Parents who reported elevated chronic stress had stress levels 0.61 higher (95% CI: (0.30, 0.93), P<0.001) than parents who reported low chronic stress. Interpersonal transient stressors had the strongest within-day effect on stress (1.45, 95% CI: (1.25, 1.64)). The strength of the across day association weakened by day two for interpersonal stressors and by day three the relationship was not found to be statistically significant*

Parents who reported overall high chronic stress had end-of-day stress levels 0.61 higher (95% CI: (0.3, 0.93), *p* = 0.002) compared to parents who reported low chronic stress (Table 1). However, there were no significant relationships between chronic stressful life events (active or resolved) reported by parents and end-of-day stress levels.

### Relationship between within- and across-day transient stressors and food-related parenting practices

With regard to the relationships between transient stressors and the types of food served at meals, interpersonal transient stressors had positive relationships with serving fast food at meals within-day (0.09; 95% CI: (0.01, 0.16), *p* = 0.031) and with serving pre-prepared foods at meals across-day (0.10; 95% CI: 0.00, 0.20), *p* = 0.049) (Table 2). Financial transient sources of stress had a strong positive relationship with serving pre-prepared foods at meals within-day (0.22; 95% CI: (0.03, 0.42), *p* = 0.026) and was negatively associated with serving homemade foods at meals across-day (−0.25; 95% CI: (−0.47, −0.02), *p* = 0.0351).

**Table 2:** Effects of chronic and lagged transient stressors on types of foods served at meals (i.e., fast food, pre-prepared food, homemade food) (*N*=61 participants; 383 observation days)^a^

	Outcome: fast food			Outcome: pre-prepared food			Outcome: homemade food		
Independent predictor variable	Mean response	95% CI	*P* value^b^	Mean response	95% CI	*P* Value	Mean response	95% CI	*P* value
**Chronic stressors**									
*High chronic stress indicator (ref: low chronic stress)*	0.08	(-0.06, 0.22)	0.237	-0.07	(-0.23, 0.10)	0.423	-0.09	(-0.24, 0.07)	0.269
*Stressful life events (prior 6 months)*									
Active (*ref: no active events*)	**-0.12**	**(-0.22, -0.01)**	**0.033**	0.00	(-0.12, 0.13)	0.939	0.08	(-0.03, 0.20)	0.159
Resolved (*ref: no resolved events*)	0.07	(-0.02, 0.17)	0.116	-0.05	(-0.16, 0.06)	0.383	-0.03	(-0.13, 0.07)	0.538
**Transient stressors**									
*Lagged sources of stress*									
*"A lot of work at home, school, or job"*									
L0. (same day)	0.06	(-0.01, 0.13)	0.070	-0.04	(-0.13, 0.04)	0.348	-0.01	(-0.10, 0.08)	0.855
L1. (second day)	0.00	(-0.07, 0.06)	0.891	0.00	(-0.08, 0.08)	0.962	0.05	(-0.04, 0.14)	0.282
L2. (third day)	0.02	(-0.05, 0.08)	0.579	0.00	(-0.08, 0.08)	0.975	-0.02	(-0.11, 0.06)	0.614
*"Conflicts with spouse, partner, or children"*									
L0. (same day)^c^	**0.09**	**(0.02, 0.17)**	**0.018**	-0.05	(-0.15, 0.05)	0.311	-0.03	(-0.13, 0.08)	0.620
L1. (second day)	-0.03	(-0.11, 0.05)	0.469	**0.10**	**(0.00, 0.20)**	**0.047**	0.00	(-0.10, 0.11)	0.964
L2. (third day)	0.02	(-0.06, 0.10)	0.621	0.01	(-0.09, 0.1)	0.899	0.01	(-0.10, 0.11)	0.902
*"Financial problems"*									
L0. (same day)	-0.02	(-0.17, 0.14)	0.808	**0.22**	**(0.03, 0.42)**	**0.027**	-0.01	(-0.22, 0.19)	0.913
L1. (second day)	0.02	(-0.15, 0.19)	0.819	-0.13	(-0.34, 0.09)	0.244	0.14	(-0.08, 0.36)	0.210
L2. (third day)	0.10	(-0.07, 0.26)	0.250	0.07	(-0.14, 0.28)	0.523	**-0.24**	**(-0.46, -0.02)**	**0.035**

^a^Model adjusted for: Parent sex, age, race, country of origin, relationship status, acculturation status (assimilation, separation, and integration), and day of the week

^b^Boldface values indicate statistical significance at *p* < 0.05

^c^ *Interpretation Example: Interpersonal transient stressors had positive relationships with fast food within-day (0.09, 95% CI: (0.02, 0.17), P=0.018)*

With regard to the relationships between transient stressors and food categories served at meals, results overall indicated that the presence of specific foods served at meals was not associated with transient stressors either within- or across-day, with some exceptions (Table 3).

**Table 3:** Effects of chronic and lagged transient stressors on daily meals serving desserts, fruits and vegetables, meat and plant protein, grains, and dairy (*N*=61 participants; 383 observation days)^a^

	Outcome: cake, candy, & SSB			Outcome: fruits and vegetables			Outcome: meat and plant protein			Outcome: grains			Outcome: dairy		
Independent predictor variable	Mean response	95% CI	*P* value^b^	Mean response	95% CI	*P* value	Mean response	95% CI	*P* value	Mean response	95% CI	*P* value	Mean response	95% CI	*P* value
**Chronic stressors**															
*High chronic stress Indicator (ref: low chronic stress)*^*c*^	0.07	(-0.08, 0.23)	0.374	**-0.18**	**(-0.35, -0.00)**	**0.048**	**-0.28**	**(-0.44, -0.11)**	**0.001**	-0.07	(-0.22, 0.08)	0.391	-0.16	(-0.34, 0.02)	0.087
*Stressful life events (prior 6 months)*															
Active (*ref: no active events*)	-0.06	(-0.18, 0.06)	0.351	0.07	(-0.07, 0.20)	0.314	**-0.16**	**(-0.29, -0.04)**	**0.011**	-0.10	(-0.22, 0.01)	0.077	**-0.16**	**(-0.3, -0.02)**	**0.028**
Resolved (*ref: no resolved events*)	0.01	(-0.10, 0.11)	0.917	**0.13**	**(0.01, 0.25)**	**0.030**	0.08	(-0.03, 0.19)	0.136	0.02	(-0.08, 0.12)	0.739	0.08	(-0.04, 0.20)	0.212
**Transient stressors**															
*Lagged sources of stress*															
*"A lot of work at home, school, or job"*															
L0. (same day)	-0.02	(-0.09, 0.06)	0.642	-0.02	(-0.10, 0.07)	0.702	-0.04	(-0.12, 0.04)	0.333	-0.06	(-0.14, 0.02)	0.154	-0.08	(-0.16, 0.01)	0.075
L1. (second day)	0.02	(-0.05, 0.10)	0.584	0.05	(-0.04, 0.13)	0.261	**0.09**	**(0.01, 0.17)**	**0.022**	-0.01	(-0.09, 0.06)	0.714	-0.03	(-0.12, 0.05)	0.435
L2. (third day)	-0.02	(-0.09, 0.05)	0.585	-0.01	(-0.09, 0.07)	0.822	0.02	(-0.06, 0.10)	0.643	0.00	(-0.08, 0.07)	0.922	0.02	(-0.06, 0.11)	0.613
*"Conflicts with spouse, partner, or children"*															
L0. (same day)	0.03	(-0.06, 0.12)	0.559	0.06	(-0.04, 0.16)	0.232	0.01	(-0.09, 0.10)	0.915	0.05	(-0.05, 0.14)	0.338	-0.03	(-0.13, 0.08)	0.625
L1. (second day)	0.06	(-0.03, 0.15)	0.170	-0.02	(-0.12, 0.08)	0.713	0.02	(-0.07, 0.11)	0.671	-0.04	(-0.14, 0.06)	0.413	0.04	(-0.07, 0.14)	0.493
L2. (third day)	-0.04	(-0.13, 0.04)	0.320	-0.01	(-0.10, 0.09)	0.895	0.07	(-0.02, 0.16)	0.109	0.04	(-0.05, 0.13)	0.409	0.06	(-0.04, 0.16)	0.235
*"Financial problems"*															
L0. (same day)	0.02	(-0.15, 0.19)	0.824	0.00	(-0.19, 0.19)	0.992	-0.06	(-0.24, 0.11)	0.471	**0.23**	**(0.05, 0.41)**	**0.010**	-0.04	(-0.23, 0.16)	0.708
L1. (second day)	-0.11	(-0.29, 0.07)	0.244	0.16	(-0.04, 0.37)	0.121	0.04	(-0.15, 0.23)	0.673	-0.10	(-0.29, 0.10)	0.336	-0.06	(-0.27, 0.15)	0.566
L2. (third day)	-0.05	(-0.24, 0.14)	0.627	-0.09	(-0.30, 0.13)	0.421	0.14	(-0.06, 0.34)	0.167	**-0.24**	**(-0.44, -0.03)**	**0.022**	0.16	(-0.06, 0.38)	0.155

^a^Model adjusted for: Parent sex, age, race, country of origin, relationship status, acculturation status (assimilation, separation, and integration), and day of the week

^b^Boldface values indicate statistical significance at *p* < 0.05

^c^ *Interpretation Example: Parents who reported high chronic stress were statistically less likely to serve fruits and vegetables (-0.18, 95% CI: (-0.35, -0.00), P=0.019) and meat and plant proteins (-0.28, 95% CI: (-0.44, -0.11)), relative to parents who experienced low chronic stress.*

With regard to the relationships between transient stressors and parent feeding practices, interpersonal conflicts with partners and children were strongly, positively related with engaging in restrictive feeding practices at meals within-day (0.09; 95% CI: (0.03, 0.16), *p* = 0.005), however there was not evidence of a persistent lag effect across-day (*p* > 0.05; Table 4). The relationship between interpersonal stress and pressure-to-eat feeding practices was unclear. The within-day and first day lag association was borderline statistically significant, but the signs were reversed. The second day lag was positively associated with pressure-to-eat feeding practices (0.06; 95% CI: (0.01, 0.12), *p* = 0.040). Financial and work, school, or home transient stressors were not found to impact parent feeding practices within- or across-days.

**Table 4:** Effects of chronic and lagged transient stressors on pressure-to-eat or restriction parent feeding practices at meals (*N*=61 participants; 383 observation days)^a^

	Outcome: pressure-to-eat feeding practices			Outcome: restriction feeding practices		
Independent predictor variable	Mean response	95% CI	*P* value^b^	Mean response	95% CI	*P* value
**Chronic stressors**						
*High chronic stress indicator (ref: low chronic stress)*	-0.12	(-0.28, 0.05)	0.161	-0.09	(-0.25, 0.07)	0.249
*Stressful life events (prior 6 months)*						
Active (*ref: no active events*)	0.03	(-0.09, 0.16)	0.632	-0.04	(-0.17, 0.08)	0.479
Resolved (*ref: no resolved events*)	**0.15**	**(0.05, 0.26)**	**0.005**	-0.01	(-0.12, 0.10)	0.856
**Transient stressors**						
*Lagged sources of stress*						
*"A lot of work at home, school, or job"*						
L0. (same day)	-0.03	(-0.08, 0.02)	0.291	0.04	(-0.02, 0.10)	0.180
L1. (second day)	-0.01	(-0.06, 0.04)	0.771	-0.02	(-0.08, 0.03)	0.391
L2. (third day)	0.01	(-0.04, 0.06)	0.755	0.00	(-0.05, 0.06)	0.926
*"Conflicts with spouse, partner, or children"*						
L0. (same day)^c^	0.05	(-0.02, 0.11)	0.147	**0.09**	**(0.03, 0.16)**	**0.005**
L1. (second day)	-0.05	(-0.12, 0.01)	0.090	0.01	(-0.06, 0.08)	0.764
L2. (third day)	**0.06**	**(0.00, 0.12)**	**0.047**	0.04	(-0.03, 0.10)	0.243
*"Financial problems"*						
L0. (same day)	-0.06	(-0.18, 0.06)	0.311	0.02	(-0.11, 0.15)	0.753
L1. (second day)	0.08	(-0.05, 0.22)	0.215	0.04	(-0.10, 0.18)	0.564
L2. (third day)	-0.01	(-0.14, 0.12)	0.901	0.06	(-0.08, 0.19)	0.422

^a^Model adjusted for: Parent sex, age, race, country of origin, relationship status, acculturation status (assimilation, separation, and integration), and day of the week

*Interpretation Example: High chronic stress over the last 30 days (indicator coded "high or low") was not statistically associated with either food pressuring or restriction* (*P* > 0.05)*. Parents with resolved stressful life events were more likely to pressure (0.15, 95% CI: (0.05, 0.26), P=0.005), and those who reported low food security (relative to the most food secure) were more likely to use restrictive feeding practices (0.32, 95% CI: (0.07, 0.56)).*

^b^Boldface values indicate statistical significance at *p* < 0.05.

^c^ *Interpersonal transient stressors (i.e., conflicts with partners and children) were strongly, positively related to the fraction of meals in which restrictive feeding practices were used within the day (0.09, 95% CI: (0.03, 0.16), P=0.005), however there was not evidence of a persistent lag effect (P>0.05)*

### Relationship between chronic stress levels and food-related parenting practices

With regard to the relationships between chronic stress and types of food served at the meal, parents who reported high chronic stress were not statistically more likely to prepare one type of food over another (*p* > 0.05; Table 2). Parents who reported active stressful life events were less likely to serve fast food for meals (−0.12; CI: (−0.22, −0.01), *p* = 0.033) compared to parents who reported no stressful life events.

With regard to the relationship between chronic stress and food categories served at meals, parents who reported elevated chronic stress levels served significantly fewer meals with fruits and vegetables (−0.18; 95% CI: (−0.35, 0.00), *p* = 0.048) and meat and plant proteins (−0.28; (−0.44, −0.11); *p* = 0.001) at meals (Table 3). Parents who reported active stressful life events also served fewer meat and plant proteins (−0.16; 95% CI: (−0.29, −0.04), 0.011) and dairy foods (−0.16; 95% CI: (−0.30, −0.02), *p* = 0.28) relative to those who reported no active stressful life events. Additionally, parents reporting resolved life events (0.13; 95% CI: (0.01, 0.25), *p* = 0.030) served more fruits and vegetables at meals relative to those with no resolved events. There were no significant associations found between chronic stressors and serving desserts/sugar sweetened beverages and any grains.

With regard to the relationships between chronic stress and parent feeding practices, high chronic stress was not statistically associated with restriction or pressure-to-eat parent feeding practices (*p* > 0.05; Table 4). There was a significant positive relationship found between resolved stressful life events and parent pressure-to-eat feeding practices (0.15; CI: (0.05, 0.26)) compared to parents reporting no stressful life events (*p* = 0.005).

## Discussion

Descriptive findings indicated that transient stressors were variable for several racial ethnic groups including, White, African American, Hispanic, and Somali families and chronic stressors were higher for African American and Native American families. These are new findings and indicate the need for addressing both transient and chronic stressors in future research. One potential hypothesis regarding why there were differences across race/ethnicity for transient stressors include that Native American and Hmong parents may not respond to transient stressors in a similar way as other parents, or may cope with transient stressors in a way that reduces the overall stress level felt.

Additionally, study findings suggested that transient and chronic stressors are associated with different food-related parenting practices (i.e., transient stressors are more influential on types of foods served and parent feeding practices; chronic stressors are more influential on food categories served), however results were not consistent. Results related to transient stress indicated that transient stressors are associated with both immediate within-day effects and persistent across-day/week effects on food-related parenting practices. Specifically, interpersonal transient stressors had immediate, strong effects on parent food-related parenting practices that wore off within a relatively short time period (e.g., within 2 days), whereas, financial transient stressors had immediate, weak impacts on parent food-related parenting practices, but lasted longer (e.g., across 2–3 days). Given that some sources of transient stress elicit strong momentary stress responses that dissipate or are resolved quickly and others have more weak immediate responses, but last longer across the week, future intervention research may want to utilize intervention tools such as ecological momentary intervention (e.g., smartphone-based messages sent to parents) techniques that can intervene on parent’s transient stressors in real-time to shape their food-related parenting practices [30].

Results related to chronic stress suggested that chronic stressors (i.e., overall chronic stress levels, stressful life events) have a strong relationship with specific food categories served at meals (e.g., fruits and vegetables), but not with types of foods served at meals (e.g., fast food, pre-prepared, homemade) or parent feeding practices (e.g., restriction, pressure-to-eat) engaged in during meals. One potential hypothesis for these findings, consistent with the Minority Stress Model [20] is that parents experiencing chronic stress such as, chronic stressful life events adapt to these stressors and it becomes their “new normal” thus, buying fast food or changing parent feeding practices in response to ongoing/chronic stress may not occur in the same way it would with transient stressors where parents opt for easier meal options such as fast food to ameliorate their stress levels. However, parents with chronic stress may serve less fruits and vegetables at meals due to the type of the ongoing chronic stress such as, unemployment where buying fruits and vegetables may cost more so they serve less of them overall at meals.

Study findings both support and extend prior research on stress and food-related parenting practices. For example, our findings corroborate prior research indicating that stress is associated with food-related parenting practices [17, 18, 30]. Additionally, study findings suggesting that transient and chronic stressors may have different relationships with food-related parenting practices (i.e., transient stressors are more influential on types of foods served and parent feeding practices; chronic stressors are more influential on food categories served) is a new finding and may indicate that transient stressors are important to target in interventions aiming to improve food-related parenting practices rather than solely focusing on chronic stressors.

There were both strengths and limitations of the current study. Strengths of the current study include the use of EMA to measure behaviors at multiple time points within and across days over an eight-day period. Additionally, EMA was used to measure both exposure (i.e., parent transient stress) and outcome variables (parent feeding practices, types of food served at meals) across time and context. Additionally, the study measured both transient and chronic sources of stress, which has not been done in prior studies on food-related parenting practices. Furthermore, the sample included racially/ethnically and socioeconomically diverse participants, as well as immigrant populations. There are also limitations of the current study, one includes the use of items from scales that have not been used with EMA or with immigrant populations. Additionally, although EMA methods allow for capturing potential fluctuations in behaviors over context and time, there could be potential reporting bias from measurement reactivity and participants in the current study could have skipped filling out mealtime event contingent surveys. Also, because the current sample was mostly from low-income households and were mothers, there is potentially lack of generalizability to higher-income groups and fathers. A further limitation is that the overall sample size was relatively small (*n* = 61) however, there were almost 400 data points used in analyses.

## Conclusions and implications for professionals

Transient sources of stress were significantly associated with food-related parenting practices in racially/ethnically and immigrant households within-day and across-day. Chronic stressors were not consistently associated with food-related parenting practices. There are potential implications for future research and development of interventions related to the current study findings. For example, it may be important for intervention materials to focus on educating parents about transient stressors and their potential effects on food-related parenting practices. Interventions may want to in particular, focus on targeting parental interpersonal conflict and financial transient stressors and provide resources to parents related to both interpersonal and financial stressors to promote healthful food-related parenting practices. Furthermore, future research may want to use intervention tools such as ecological momentary intervention (e.g., smartphone-based messages sent to parents) techniques that can intervene on parent’s transient stressors in real-time to shape their food-related parenting practices.
